# A Diagnostic Challenge: Necrotizing Retropharyngeal Lymphadenitis With Microbiologically Supported Multifocal Pseudomonas aeruginosa Infection and a Tularemia-Like Presentation

**DOI:** 10.7759/cureus.115453

**Published:** 2026-08-30

**Authors:** Sajjad M AlKadhem, Ali T Alamer, Osama Kattih, Ahmed A Al-Amoudi, Zainab A Almoosa, Aisha M Shathele

**Affiliations:** 1 Pediatric Intensive Care Unit, Almoosa Specialist Hospital, Al-Ahsa, SAU; 2 Pediatrics, Almoosa Specialist Hospital, Al-Ahsa, SAU; 3 Pediatric Infectious Diseases, Almoosa Specialist Hospital, Al-Ahsa, SAU

**Keywords:** deep neck infection, facial nerve palsy, necrotizing lymphadenitis, pediatric septic shock, pseudomonas aeruginosa, tularemia

## Abstract

Necrotizing retropharyngeal lymphadenitis can cause rapidly progressive airway compromise and systemic illness in young children. We describe a two-year-old boy with no previously recognized chronic disease or immune disorder who presented with fever, stridor, hypoxemia, cytopenias, markedly elevated inflammatory markers, and a necrotic right-cheek lesion. Progressive airway compromise required endotracheal intubation, and contrast-enhanced computed tomography revealed bilateral necrotic retropharyngeal lymph nodes or collections with surrounding edema, as well as cervical lymphadenopathy. He subsequently developed septic shock with respiratory, cardiovascular, and coagulation dysfunction. A household rabbit had died four days before presentation, raising concern for tularemia, although a bite or scratch could not be confirmed. Tularemia serology obtained within the first week of illness was negative and was not repeated; therefore, tularemia could not be definitively excluded. During admission, additional papulopustular skin lesions and otorrhea developed. *Pseudomonas aeruginosa* was isolated from ear discharge, a deep tracheal aspirate, and a pustular skin lesion, supporting a clinically significant multifocal infection at the sampled sites. Blood cultures remained negative, and the retropharyngeal lesions were not sampled; therefore, their microbiological etiology could not be established. The child improved with antimicrobial therapy and multidisciplinary management. This case highlights the importance of distinguishing microbiologically confirmed infection from anatomically plausible but unproven causal associations when epidemiologic, radiologic, and microbiological findings do not fully align.

## Introduction

Deep neck infections in children can progress from lymphadenitis or phlegmon to suppuration, airway obstruction, sepsis, or extension into adjacent spaces. Contrast-enhanced computed tomography helps define the anatomical extent and radiological maturity of the process. However, the decision to pursue surgical drainage should integrate airway status, rim enhancement, lesion anatomy, clinical trajectory, and the feasibility of close reassessment rather than relying on lesion size alone [1,2].

Severe community-acquired *Pseudomonas aeruginosa* infection is uncommon in children without previously recognized disease. Reported pediatric presentations include leukopenia or neutropenia, thrombocytopenia, pustular or necrotic skin lesions, otitis or mastoid involvement, pneumonia, shock, and cranial nerve complications [3,4]. Tularemia may produce an overlapping phenotype when rabbit exposure, an inoculation ulcer, and suppurative cervical lymphadenitis are present [5]. Early serology, however, has limited sensitivity and cannot reliably exclude infection during the first week of illness [6,7].

We report a child with airway compromise, bilateral necrotizing retropharyngeal lymphadenitis, septic shock, papulopustular skin lesions, otorrhea, mastoid changes, and *Pseudomonas aeruginosa* recovered from multiple clinically involved sites after recent rabbit exposure. The case highlights the diagnostic challenge created by overlapping clinical, epidemiologic, and microbiological features of tularemia and severe *Pseudomonas aeruginosa* infection.

## Case presentation

A two-year-old boy weighing 12 kg was brought to the pediatric emergency department after two days of fever, respiratory distress, reduced oral intake, and noisy breathing. He had no previously recognized chronic disease or diagnosed immune disorder. There was no history of recurrent or unusually severe infections and no prior antibiotic exposure. He was well developed for his age, with no documented growth or developmental concerns. There was no known family history of immunodeficiency. Newborn screening had reportedly been normal, and immunizations were up to date for age. On arrival, he appeared acutely ill. His temperature was 39.8°C, heart rate was 168 beats/min, respiratory rate was 48 breaths/min, blood pressure was 96/58 mmHg, oxygen saturation was 88% on room air, and capillary refill time was approximately two seconds. Examination revealed marked inspiratory stridor, intercostal and subcostal retractions, increased work of breathing, and a necrotic lesion on the right cheek. He was admitted to the pediatric intensive care unit.

Admission investigations showed leukopenia with neutropenia, thrombocytopenia, markedly elevated inflammatory markers, and a mildly elevated blood lactate concentration. Cerebrospinal fluid contained no white or red blood cells, and blood and cerebrospinal fluid cultures were negative (Table 1). Leukopenia, neutropenia, and thrombocytopenia improved progressively over the subsequent clinical course and later normalized. Given the spontaneous recovery of the cytopenias and clinical improvement, bone marrow aspiration was deemed clinically unnecessary and was not performed. Chest radiography demonstrated an upper-lobe airspace opacity (Figure 1). Abdominal ultrasonography was interpreted as showing mild splenomegaly without a focal lesion. The kidneys appeared normal.

**Table 1:** Admission laboratory findings

Test	Patient result	Interpretation
White blood cell count	2.2 × 10⁹/L	Low
Absolute neutrophil count	0.7 × 10⁹/L	Low
Hemoglobin	10.6 g/dL	Within the reported pediatric interval
Platelet count	115 × 10⁹/L	Low
C-reactive protein	413 mg/L	Markedly elevated
Procalcitonin	63.2 ng/mL	Markedly elevated
Erythrocyte sedimentation rate	92 mm/h	Elevated
Blood lactate	2.4 mmol/L	Mildly elevated
Cerebrospinal fluid cells	0 white cells; 0 red cells	No pleocytosis
Cerebrospinal fluid glucose	4.68 mmol/L	No paired serum glucose was available for comparison
Cerebrospinal fluid protein	147 mg/L (14.7 mg/dL)	Within the reported pediatric interval

**Figure 1:**
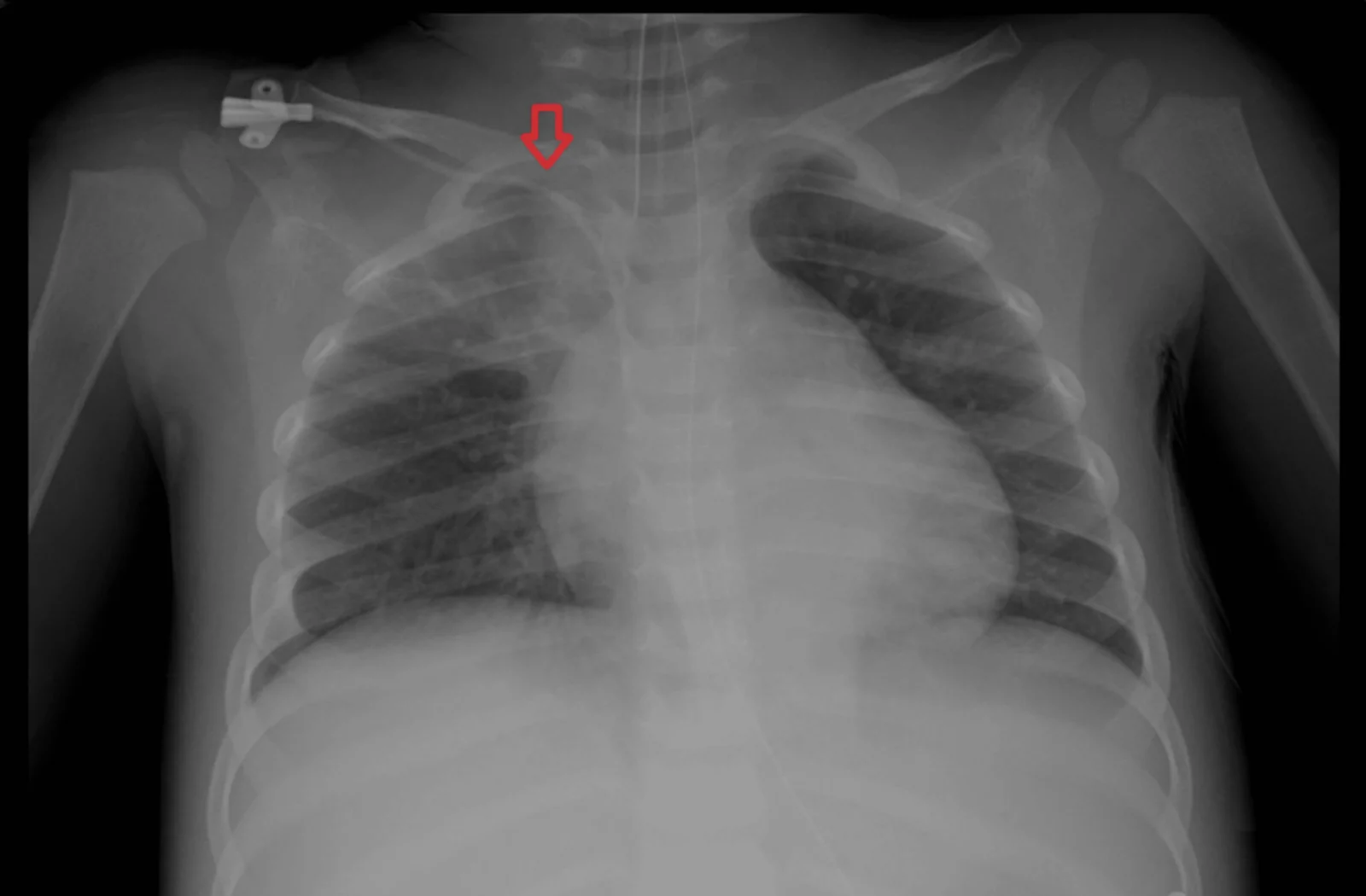
Chest radiograph demonstrating a right upper-lobe airspace opacity

Persistent stridor and clinical deterioration led to endotracheal intubation and invasive ventilation on hospital day three. Contrast-enhanced computed tomography of the neck on hospital day three demonstrated bilateral necrotic retropharyngeal nodal lesions or collections, measuring approximately 25 × 10 mm on the right and 26 × 9 mm on the left, with surrounding retropharyngeal soft-tissue edema and multiple enlarged cervical lymph nodes (Figure 2). The radiological appearance was considered consistent with necrotizing retropharyngeal lymphadenitis. The available report did not describe a well-defined rim-enhancing cavity, a measurable central nonenhancing fluid component, mediastinal extension, or other features confirming a mature drainable abscess. Accordingly, the lesions are described in this report as necrotizing retropharyngeal lymphadenitis rather than definitively as retropharyngeal abscesses.

**Figure 2:**
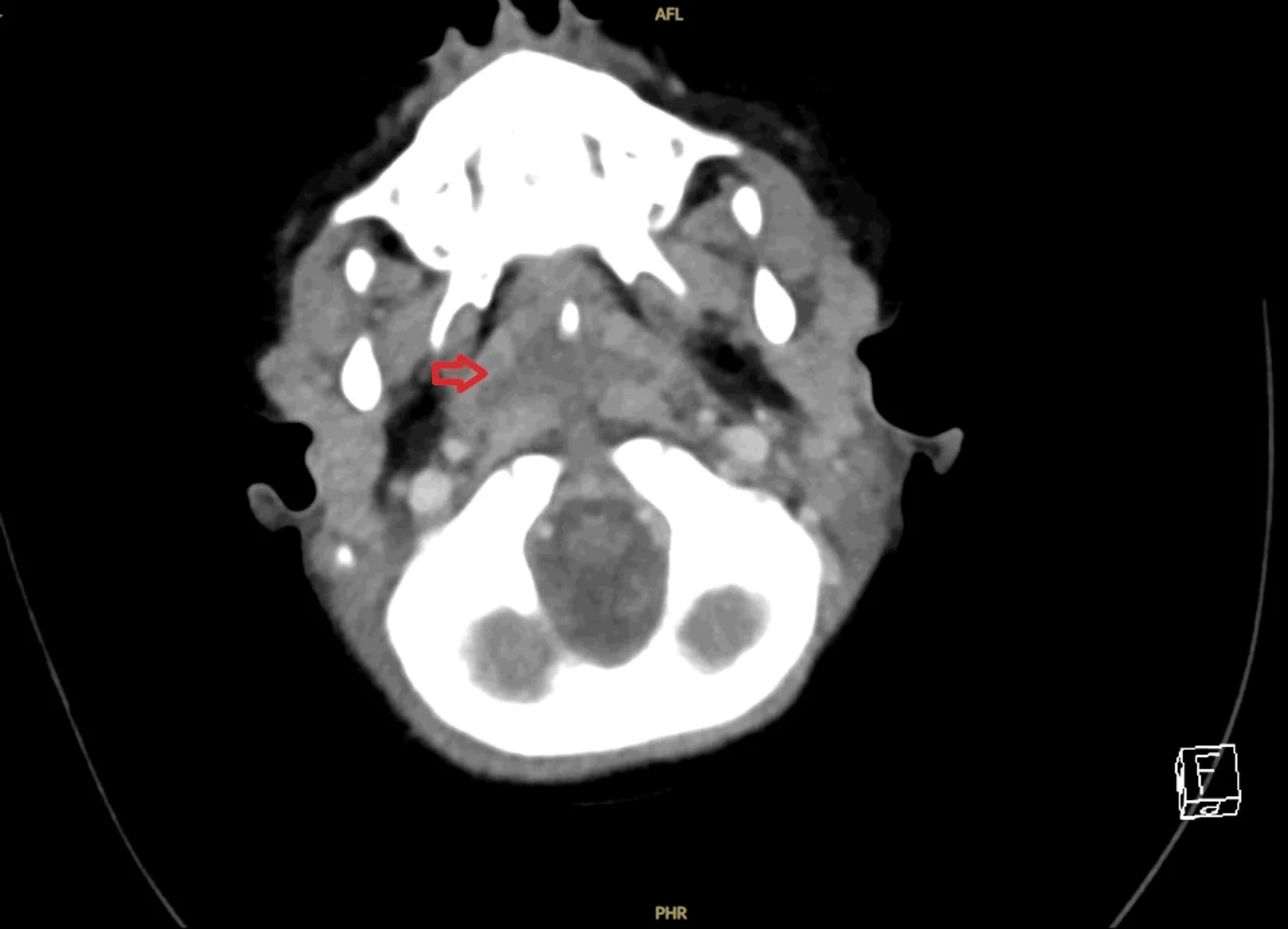
Contrast-enhanced CT of the neck on hospital day 3 demonstrating bilateral necrotic retropharyngeal lymph nodes with surrounding soft-tissue edema and cervical lymphadenopathy

During hospital days three and four, the child developed infection-associated respiratory, cardiovascular, and coagulation dysfunction. The lowest documented blood pressure was 50/39 mmHg, with a mean arterial pressure of 43 mmHg. Hypotension required epinephrine for two days, titrated to a maximum of 0.08 µg/kg/min, and was weaned as hemodynamic stability improved. An eight-hour period of anuria resolved after furosemide without subsequent documented kidney injury. Transthoracic echocardiography, obtained during febrile hemodynamic deterioration because infective endocarditis was considered, was normal and showed no visible valvular vegetation.

Otolaryngology, pediatric infectious disease, and pediatric intensive care teams jointly reviewed the need for operative drainage. In view of the radiological uncertainty regarding a mature abscess, the child’s evolving clinical response, and the ability to provide close airway monitoring and repeat imaging, the teams selected medical management with serial reassessment.

Further history revealed that a household rabbit had become ill and died four days before the child’s presentation. The family was uncertain whether the child had been bitten or scratched. The necrotic right-cheek lesion present at admission was considered compatible with a possible inoculation lesion, and the combination of rabbit exposure, the cutaneous lesion, and necrotizing cervical lymphadenitis raised concern for tularemia.

A single serological test for Francisella tularensis immunoglobulin M and immunoglobulin G, obtained on hospital day three and within the first week of illness, was negative. No convalescent serology, *Francisella tularensis* polymerase chain reaction, culture, lymph-node aspiration, or tissue examination was performed. Because antibody responses are often absent early in the illness, this result did not reliably rule out tularemia.

Additional erythematous papules appeared during admission and progressed to pustules on the face, trunk, back, and lower extremities (Figure 3). Herpes simplex virus infection was considered because of the evolving skin lesions. Serum herpes simplex virus immunoglobulin G was positive, but lesion, blood, and cerebrospinal fluid polymerase chain reaction testing was negative. A repeat lumbar puncture after intubation again showed no evidence of central nervous system infection.

**Figure 3:**
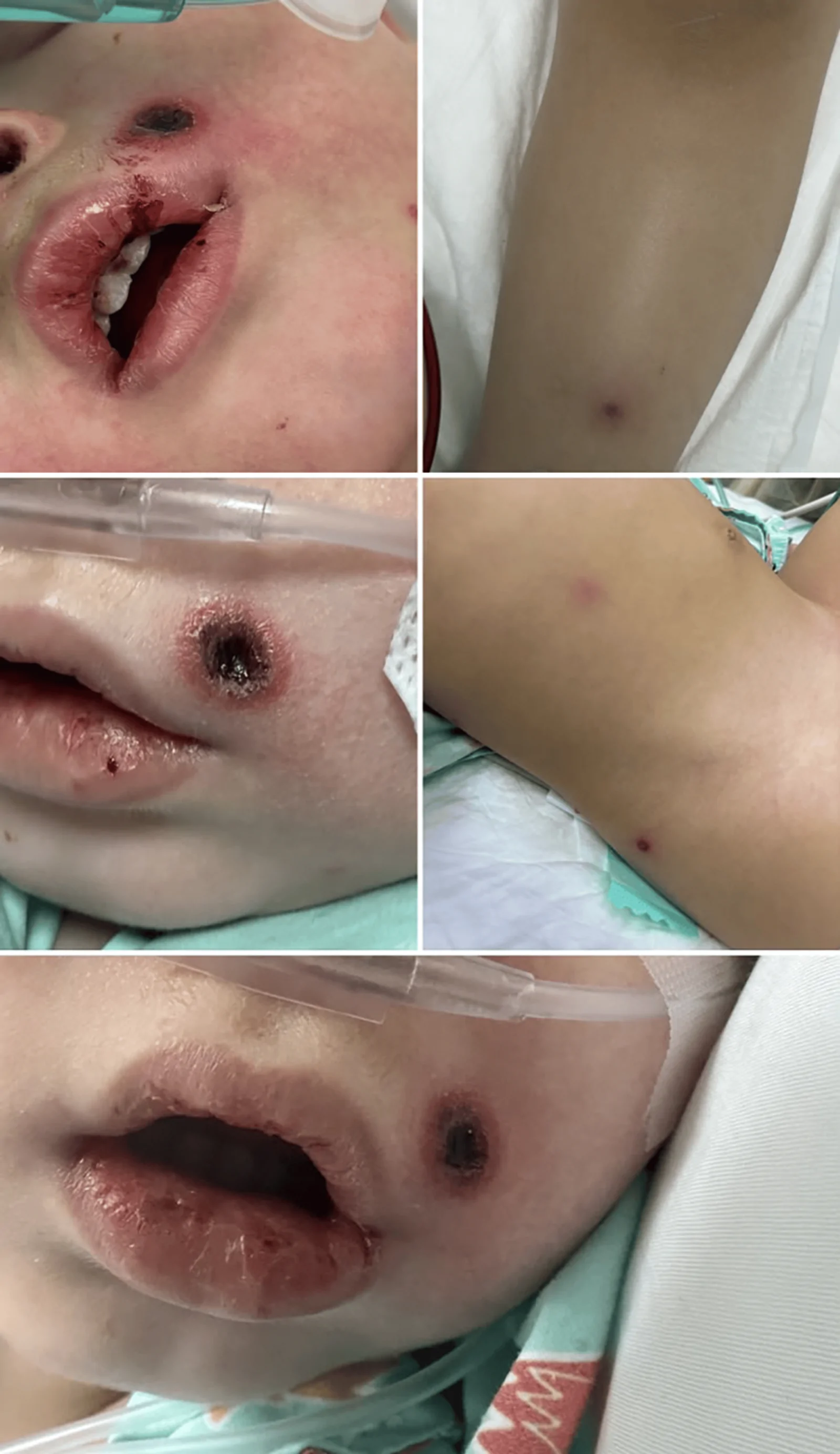
Necrotic right-cheek lesion present at admission and subsequent evolution of additional cutaneous lesions from erythematous papules to pustules involving the face, trunk, back, and lower extremities

Otorrhea developed on hospital day five. *Pseudomonas aeruginosa* was isolated from ear discharge, a deep tracheal aspirate, and a pustular skin lesion. The tracheal isolate was reported as susceptible to all antimicrobials tested. The isolates were not molecularly typed, and the exact collection dates for the tracheal and skin specimens and their timing relative to antipseudomonal therapy were not fully available for review. Blood cultures remained negative throughout the admission.

Intravenous ceftriaxone was started after the initial sepsis evaluation for empirical coverage of sepsis and deep-neck infection. With subsequent clinical deterioration, therapy was broadened to include meropenem for Gram-negative coverage with antipseudomonal activity, and vancomycin for empirical coverage of methicillin-resistant Staphylococcus aureus. Clindamycin was added because toxin-mediated bacterial disease remained a consideration. Acyclovir was started while disseminated herpes simplex virus infection was being evaluated. After the history of recent rabbit exposure, the necrotic cheek lesion, and necrotizing cervical lymphadenitis raised concern for severe tularemia; gentamicin was added on hospital day three, providing additional antipseudomonal activity. Gentamicin was administered for 10 days. The anti-infective courses and available clinical rationales are summarized in Table 2.

**Table 2:** Antimicrobial treatment

Agent	Start	Duration	Clinical rationale
Ceftriaxone	Day 0	One day	Initial empirical therapy for sepsis and deep-neck infection; replaced when clinical deterioration prompted broader coverage
Meropenem	Day 1	14 days	Broad Gram-negative, antipseudomonal, and deep-neck infection coverage
Vancomycin	Day 1	10 days	Empirical coverage for methicillin-resistant Staphylococcus aureus during severe systemic illness
Clindamycin	Day 1	Five days	Added while toxin-mediated bacterial disease remained a diagnostic consideration
Gentamicin	Day 3	10 days	Added for suspected severe tularemia after rabbit exposure and compatible clinical findings; also provided additional antipseudomonal activity
Acyclovir	Day 1	10 days	Empirical treatment while disseminated herpes simplex virus infection was being evaluated

Repeat contrast-enhanced computed tomography on hospital day 10 showed interval reduction in the retropharyngeal fluid and cervical lymphadenopathy, with a residual left peritonsillar collection measuring 1.3 × 0.9 × 1.3 cm (Figure 4). The report also described complete opacification of the left mastoid air cells and middle-ear cavity and new partial opacification of the right mastoid air cells, without documented bony erosion or coalescent mastoiditis. Pulmonary airspace disease had improved substantially.

**Figure 4:**
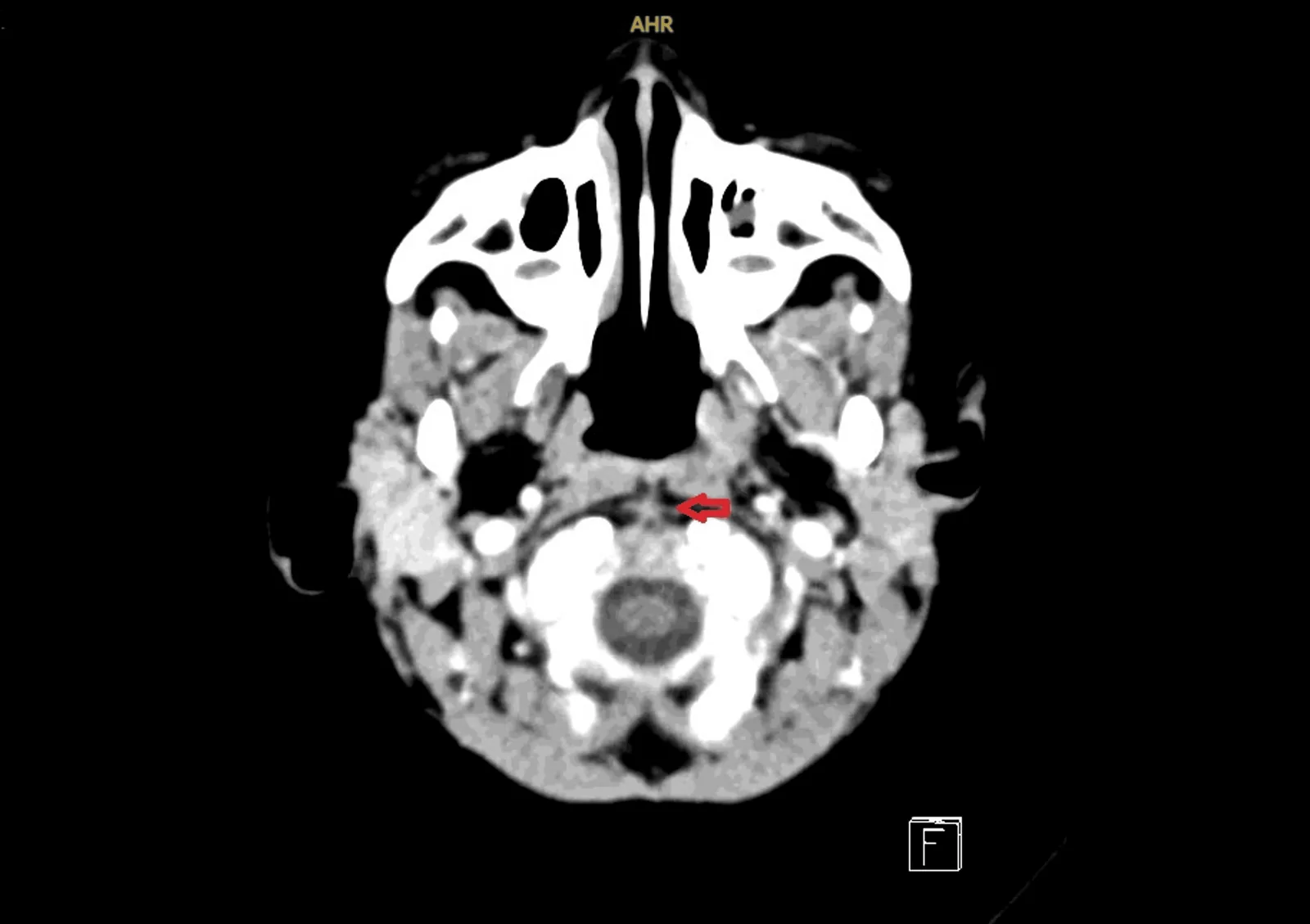
Follow-up contrast-enhanced CT on hospital day 10 demonstrating significant interval regression of the bilateral retropharyngeal nodal abnormalities and mastoid air-cell opacification CT: computed tomography

Respiratory status and inflammatory markers improved progressively, and the child was extubated and received oxygen via nasal cannula on hospital day 12. Computed tomography of the brain after extubation showed no acute intracranial abnormality. A right facial nerve palsy was recognized after extubation and treated with intravenous methylprednisolone for 10 days. The available records did not include details of the steroid dose or taper, standardized facial-nerve grading, formal hearing assessment, or the exact follow-up interval. Facial function improved during pediatric neurology follow-up and was documented as fully recovered at the latest assessment.

A formal evaluation for an inborn error of immunity was not completed during the critical illness. Quantitative immunoglobulins, lymphocyte subsets, neutrophil oxidative-burst testing, human immunodeficiency virus testing, complement screening, immunology consultation, and convalescent immune testing were therefore unavailable. The overall clinical course is summarized in Table 3.

**Table 3:** Clinical timeline

Time	Event	Clinical details
Day 0	Emergency presentation	Fever, stridor, respiratory distress, hypoxemia, poor intake, and a necrotic right-cheek lesion; pediatric intensive care admission and ceftriaxone
Day 3	Airway deterioration and imaging	Endotracheal intubation; neck computed tomography showed bilateral necrotic retropharyngeal abnormalities; tularemia serology was obtained
Days 3-4	Hemodynamic deterioration	Respiratory, cardiovascular, and coagulation dysfunction; epinephrine was required for two days
During hospitalization	Evolving systemic phenotype	Rabbit exposure identified; additional papules progressed to pustules; mild splenomegaly documented
During the subsequent hospital course	Source-control review	Otolaryngology, pediatric infectious disease, and intensive care teams selected medical management with serial reassessment
Day 5 and subsequent hospital course	Microbiology	Otorrhea developed; *Pseudomonas aeruginosa* isolated from ear discharge, deep tracheal aspirate, and a pustular lesion; blood and cerebrospinal fluid cultures remained negative
Day 10	Repeat imaging	Retropharyngeal abnormalities and cervical lymphadenopathy regressed; mastoid and middle-ear opacification was reported
Day 12	Respiratory recovery	Successful extubation to oxygen by nasal cannula
Day 12	Neurological complication	Right facial nerve palsy recognized; methylprednisolone administered for 10 days
At latest follow-up	Outcome	Facial nerve function documented as fully recovered at the latest assessment

## Discussion

This case demonstrates the diagnostic difficulty that can arise when epidemiologic clues and microbiological findings suggest competing infectious etiologies. The strongest microbiological evidence supported clinically significant *Pseudomonas aeruginosa* infection, with the organism recovered from ear discharge, a pustular skin lesion, and a deep tracheal aspirate in the setting of otorrhea, evolving papulopustular skin lesions, pulmonary infiltrates, cytopenias, and septic shock. Severe community-acquired *Pseudomonas aeruginosa* infection in previously healthy children is uncommon. However, it has been associated with leukopenia or neutropenia, thrombocytopenia, pneumonia, otologic or mastoid disease, necrotic or pustular skin lesions, shock, and cranial nerve complications [3,4]. Nevertheless, blood cultures remained negative, the isolates were not molecularly typed, and the retropharyngeal lesions were not sampled. The microbiological findings therefore support *Pseudomonas aeruginosa* infection at multiple clinically involved sampled sites but do not establish bacteremia, hematogenous dissemination, or direct Pseudomonas infection of the retropharyngeal lesions.

Tularemia remained an important alternative diagnosis because of recent household rabbit exposure, a necrotic cheek lesion compatible with a possible inoculation site, and associated necrotizing cervical lymphadenitis [5]. The negative *Francisella tularensis* serology was obtained during the first week of illness, when antibodies may still be undetectable, and therefore could not reliably exclude tularemia [6,7]. No convalescent serology, *Francisella tularensis*-specific polymerase chain reaction, culture, lymph-node aspiration, or tissue examination was subsequently performed. Furthermore, clinical improvement could not reliably distinguish between the competing diagnoses because the child received both broad antipseudomonal therapy and gentamicin, which is recommended for severe tularemia [8]. The case is therefore best interpreted as a tularemia-like presentation, with microbiological evidence of *Pseudomonas aeruginosa* infection at the sampled sites. At the same time, tularemia remained unconfirmed and could not be definitively excluded.

The decision to manage the retropharyngeal lesions without operative drainage was based on the overall radiological and clinical assessment rather than lesion dimensions alone. Computed tomography demonstrated bilateral necrotic retropharyngeal abnormalities with surrounding soft-tissue edema and cervical lymphadenopathy. However, the available radiology report did not consistently demonstrate features indicative of a mature, drainable abscess, such as a well-defined rim-enhancing cavity. Contemporary pediatric data suggest that radiological characteristics, particularly rim enhancement and abscess maturity, may be more informative in predicting successful surgical drainage than lesion size or clinical symptoms alone and that selected children can be successfully managed with antimicrobial therapy and close observation [1,2]. In this patient, multidisciplinary assessment by otolaryngology, pediatric infectious disease, and intensive care teams, together with the ability to maintain a secure airway, closely monitor clinical progression, and obtain repeat imaging, supported an initial conservative strategy. Subsequent radiological regression of the retropharyngeal abnormalities without drainage supported the appropriateness of this individualized approach. However, this outcome should not be interpreted as establishing a general size threshold for nonoperative management or identifying the causative organism of the retropharyngeal process.

The severity of illness, including infection-associated organ dysfunction, invasive respiratory support, and hypotension requiring epinephrine, was clinically consistent with pediatric septic shock [9]. The leukopenia, neutropenia, and thrombocytopenia subsequently improved and normalized during clinical recovery. Although this course is compatible with transient infection-associated cytopenias, the unusual severity of community-acquired *Pseudomonas aeruginosa* infection in a child without a previously recognized underlying disorder appropriately raises consideration of an underlying predisposition. A comprehensive evaluation for an inborn error of immunity was not completed, and therefore an underlying immune abnormality cannot be definitively excluded.

The principal limitations of this report are (1) the absence of direct microbiological sampling of the retropharyngeal lesions, (2) the lack of confirmatory testing for tularemia with convalescent serology or organism-specific molecular or tissue-based methods, (3) the absence of molecular strain typing of the *Pseudomonas aeruginosa* isolates, (4) the incomplete evaluation for an underlying inborn error of immunity, and (5) the lack of standardized otologic and facial-nerve follow-up. These limitations restrict definitive attribution of the retropharyngeal process to a single pathogen and reinforce the need to distinguish microbiologically established findings from plausible but unproven causal relationships.

Overall, the clinical significance of this case lies in the need to integrate epidemiologic exposure, targeted microbiological sampling, radiological characteristics, and longitudinal clinical response when the available evidence does not support a single definitive etiologic explanation. In such circumstances, early airway stabilization, multidisciplinary reassessment of source control, appropriate empirical antimicrobial coverage, and transparent communication of diagnostic uncertainty remain central to clinical management.

## Conclusions

This case highlights the diagnostic challenge of reconciling a tularemia-like clinical presentation with microbiological evidence supporting *Pseudomonas aeruginosa* infection at multiple sampled sites. However, because the retropharyngeal lesions were not directly sampled and tularemia was not definitively excluded, the etiology of the deep-neck process could not be established with certainty. Careful distinction between microbiologically confirmed findings and plausible but unproven causal relationships is essential in complex infectious presentations.

## References

[1] Darawish SM, Shahid MA, Aziz N (2026). Deep neck space infections in children: peritonsillar, retropharyngeal, parapharyngeal, and Ludwig's angina emergencies in the pediatric emergency department. Am J Otolaryngol.

[2] Liu Y, Nicotera DJ, Islam AA, Dunsky K, Lieu JE (2024). Prognostic factors for retropharyngeal abscess in children receiving surgery or antibiotic therapy. Laryngoscope.

[3] Huang YC, Lin TY, Wang CH (2002). Community-acquired Pseudomonas aeruginosa sepsis in previously healthy infants and children: analysis of forty-three episodes. Pediatr Infect Dis J.

[4] Azapağası E, Öz FN, Uysal Yazıcı M, Ceylan D, Ocak E, Taşçı Yıldız Y, Aytekin C (2021). Pseudomonas aeruginosa sepsis in a previously healthy infant with subcutaneous nodules and mastoid bone destruction. J Pediatr Intensive Care.

[5] Maurin M, Gyuranecz M (2016). Tularaemia: clinical aspects in Europe. Lancet Infect Dis.

[6] (2026). Clinical testing and diagnosis for tularemia. https://www.cdc.gov/tularemia/hcp/diagnosis-testing/index.html.

[7] Maurin M (2020). Francisella tularensis, tularemia and serological diagnosis. Front Cell Infect Microbiol.

[8] Nelson CA, Meaney-Delman D, Fleck-Derderian S, Winberg J, Mead PS (2025). Tularemia antimicrobial treatment and prophylaxis: CDC recommendations for naturally acquired infections and bioterrorism response - United States, 2025. MMWR Recomm Rep.

[9] Schlapbach LJ, Watson RS, Sorce LR (2024). International consensus criteria for pediatric sepsis and septic shock. JAMA.

